# Adolescent Intermittent Alcohol Exposure: Deficits in Object Recognition Memory and Forebrain Cholinergic Markers

**DOI:** 10.1371/journal.pone.0140042

**Published:** 2015-11-03

**Authors:** H. Scott Swartzwelder, Shawn K. Acheson, Kelsey M. Miller, Hannah G. Sexton, Wen Liu, Fulton T. Crews, Mary-Louise Risher

**Affiliations:** 1 Durham VA Medical Center, Durham, North Carolina, 27705, United States of America; 2 Department of Psychiatry and Behavioral Sciences, Duke University Medical Center, Durham, North Carolina, 27705, United States of America; 3 Department of Psychology and Neuroscience, Duke University Medical Center Durham, Durham, North Carolina, 27705, United States of America; 4 Bowles Center for Alcohol Studies, University of North Carolina, Chapel Hill, North Carolina, 27514, United States of America; Roma Tre University, ITALY

## Abstract

The long-term effects of intermittent ethanol exposure during adolescence (AIE) are of intensive interest and investigation. The effects of AIE on learning and memory and the neural functions that drive them are of particular interest as clinical findings suggest enduring deficits in those cognitive domains in humans after ethanol abuse during adolescence. Although studies of such deficits after AIE hold much promise for identifying mechanisms and therapeutic interventions, the findings are sparse and inconclusive. The present results identify a specific deficit in memory function after AIE and establish a possible neural mechanism of that deficit that may be of translational significance. Male rats (starting at PND-30) received exposure to AIE (5g/kg, i.g.) or vehicle and were allowed to mature into adulthood. At PND-71, one group of animals was assessed using the spatial-temporal object recognition (stOR) test to evaluate memory function. A separate group of animals was used to assess the density of cholinergic neurons in forebrain areas Ch1-4 using immunohistochemistry. AIE exposed animals manifested deficits in the temporal component of the stOR task relative to controls, and a significant decrease in the number of ChAT labeled neurons in forebrain areas Ch1-4. These findings add to the growing literature indicating long-lasting neural and behavioral effects of AIE that persist into adulthood and indicate that memory-related deficits after AIE depend upon the tasks employed, and possibly their degree of complexity. Finally, the parallel finding of diminished cholinergic neuron density suggests a possible mechanism underlying the effects of AIE on memory and hippocampal function as well as possible therapeutic or preventive strategies for AIE.

## Introduction

Nearly four decades ago Donald Walker and his group showed that months of chronic ethanol exposure during adulthood resulted in enduring deficits in learning and memory [1] that were accompanied by changes in hippocampal neuronal morphology [2] and cell loss [3]. Those initial studies sparked a line of research that has had a major impact on the alcohol research field in general and has had both mechanistic and clinical implications. More recently, as it has become clear that adolescence is a time of distinctive sensitivity to the acute effects of ethanol [4–8], the question of whether repeated exposure to ethanol during adolescence results in enduring learning and memory impairments has begun to be addressed.

Initial studies found that adolescent intermittent ethanol exposure (AIE) did not alter subsequent spatial reference memory learning in the radial arm maze [9], or learning in the Barnes maze [10]. However, AIE did increase the susceptibility of adult animals pre-exposed to AIE to the memory disrupting effects of acute ethanol [9,11]. Consistent with the lack of effect of AIE on learning, comparable exposure to chronic intermittent ethanol in adulthood (CIE) also did not disrupt subsequent spatial learning [9]. However, in contrast to the effect of AIE, CIE failed to increase subsequent responsiveness to acute ethanol. Thus, while neither AIE nor CIE influenced subsequent spatial learning in the radial arm maze, AIE enhanced subsequent sensitivity to the mnemonic effects of acute ethanol, whereas CIE did not, suggesting that AIE produced enduring effects that CIE did not. Both the enhancement of subsequent memory disruption by AIE and its lack of effect on baseline spatial learning in the radial arm maze were replicated in a recent study [11]. In contrast to the lack of effect of AIE or CIE on learning in the radial arm maze, AIE has been shown to impair learning in the Morris water maze up to 25 days after then end of AIE exposure [12], and Broadwater and Spear [13] have observed deficits in fear retention at a similar time interval after AIE, but not after CIE. Earlier ethanol exposure, spanning the late juvenile period and early adolescence, has also been shown to induce deficits in object recognition memory and discrimination learning at approximately three weeks after the termination of ethanol exposure [14]. With respect to subsequent sensitivity to the memory-impairing effects of ethanol, Silvers and colleagues [15,16] found that AIE reduced the efficacy with which acute ethanol impaired spatial learning in the water maze 24 hours after the last ethanol dose, though that effect must be interpreted in light of possible withdrawal and/or tolerance effects that would be expected at that time after AIE. Most recently, it has been shown that AIE impairs adult learning on a novel object recognition task when a long delay (24 hours) was imposed between initial exposure to the novel object and retrieval testing [17]. Thus, AIE appears to impair learning and memory in adulthood, but not uniformly across dependent measures.

In this regard it is notable that in instances where animals pre-exposed to AIE were challenged, either with long inter-trial intervals [17,18] or acute ethanol treatment [9,11], deficits were observed relative to controls. This suggests that AIE may not induce a state of markedly compromised learning capacity on nominally complex or challenging tasks, but when task complexity is increased or other challenges to memory-related CNS function are introduced, AIE-induced deficits may be unmasked. Therefore, part of our rationale for the present study was to use a task that involved both spatial and temporal memory components to determine if either was more vulnerable to the effects of AIE than the other. In addition, because the stOR test alters both spatial and temporal cues in parallel within the animals’ environment, we hypothesized that this environmental complexity would be more challenging to AIE animals than controls. We predicted therefore that AIE would result in memory deficits using this task even with relatively brief inter-trial intervals relative to the longer interval at which previous studies have demonstrated AIE-induced deficits in object recognition [17,18].

In addition to the behavioral effects of AIE, recent findings indicate that AIE causes a reduction in the density of cholinergic neurons in the medial septum and the vertical limb of the diagonal band of Broca (also known as Ch1-2) [19,20]. Hippocampal circuit excitability and memory-related hippocampal functions are driven by those cholinergic inputs, suggesting that AIE may lead to chronic deprivation of cholinergic input to the hippocampal formation, which would be expected to alter both memory and memory-related hippocampal structure and function. Interestingly, unlike general lesions of Ch1-2, selective cholinergic lesions often do not result in robust learning deficits unless complex or challenging tasks are used (see Kanju et al., [21]). Thus it may be that the lack of effect of AIE on most measures of spatial memory (but see Sircar and Sircar [12]) could be related to either their relative ease across multiple days of testing, lack of task complexity, and/or their reliance on procedural components that might not be affected by moderate depletion of cholinergic hippocampal inputs.

To begin to address this, we used an adaptation of the novel object recognition task that involves both spatial and temporal memory components and does not rely on procedural training, to assess the long-term effect of AIE on spatial memory. In addition, we assessed the density of cholinergic neurons in Ch1-4 using choline acetyltransferase (ChAT) immunohistochemistry. In addition to Ch1-2, which project to the hippocampus, we also included areas Ch-3 and Ch-4 in the analysis. Ch-3 projects to the pyriform and entorhinal cortices (in addition to the olfactory bulbs) and thus would be expected to regulate hippocampal function, whereas Ch-4 projects widely across the neocortex as well as to the basolateral amygdala. Thus a comparison of the effects of AIE on ChAT immunohistochemistry across those regions will provide potentially meaningful comparisons.

## Materials and Methods

All of the procedures used in this study were conducted in accordance with the guidelines of the American Association for the Accreditation of Laboratory Animal Care and the National Research Council’s Guide for Care and Use of Laboratory Animals and were approved by the Durham VA Medical Center and the Duke University Animal Care and Use Committees. All animals used were male rats of the Sprague-Dawley strain.

Thirty-six rats (Charles River, USA) were double housed and maintained in a temperature- and humidity-controlled room with *ad libitum* access to food and water. Animals were dosed using modified methods previously described in Risher et al., [11]. Briefly, animals were delivered at PND-25 and allowed to acclimatize for 5 days in the vivarium on a reverse 12:12-hr light:dark cycle (lights off at 9:00 am) prior to beginning AIE (adolescent intermittent ethanol) or saline administration on PND-30. All animals were exposed to an AIE or saline exposure regimen beginning PND-30, consisting of 10 doses of 5 g/kg ethanol (35% v/v in saline at 18.12 mL/kg, VWR, Suwanee, GA, USA) or isovolumetric saline administered by intragastric gavage using a 2 days on, 1 day off, 2 days on, 2 days off intermittent schedule for 16 days followed by a 25-day washout period, thus allowing all animals to reach adulthood prior to sacrifice or behavioral testing. These ethanol doses were selected in order to produce BECs that are consistent with adolescent human BECs during binge drinking episodes. In a recent study, we found that animals receiving 5g/kg ethanol (i.g.) achieved average blood ethanol concentrations of 199.7mg/dl (± 19.9) 60 minutes after the first dose, and 172.8 (± 13.3) 60 minutes after the last dose [22]. Those animals were treated in parallel with those described in the present experiments. Moreover, those blood ethanol concentrations are consistent with those achieved in our earlier studies [23], and by adolescent humans during binge drinking episodes [24].

Previous studies have suggested that AIE selectively promotes adolescent-like characteristics into adulthood [25], therefore we conducted a second stOR experiment with a separate cohort of animals to compare treatment-naïve adult and adolescent animals in the same task. A total of 24 male rats (PND-24 (adolescent) and PND-64 (adult), n = 12/age group) were double housed and maintained in a temperature- and humidity-controlled room with *ad libitum* access to food and water. These animals were handled 5 times across three days to habituate the animals to the experimenter and received no gavage.

### Spatial-Temporal Object Recognition (stOR)

We used a modified version of the spatial-temporal object recognition (stOR) task described by Kart-Teke et al. [26] to assess object memory in rats exposed to AIE and tested in adulthood. This task requires no external motivation, reward, punishment, or training while still allowing us to measure the memory for spatial locations [27] and object recency [28] by using the animals’ ability to recognize and differentiate between objects [29]. This task also relies on a rodent’s innate interest in exploring novelty, be it objects in novel locations or objects novel in appearance (the latter serving as the basis for traditional novel object recognition tests). Spatial memory is inferred by the relative preference for objects in a novel location compared to a familiar location. That is, any demonstrable preference for objects in the novel location requires that animals recall the familiar location from a previous trial. Temporal memory is inferred by the relative preference for objects that have not been seen recently (2 hours since last encounter) when presented with objects that have been seen more recently (1 hour since last encounter).

#### Apparatus and habituation

**Fig 1:**
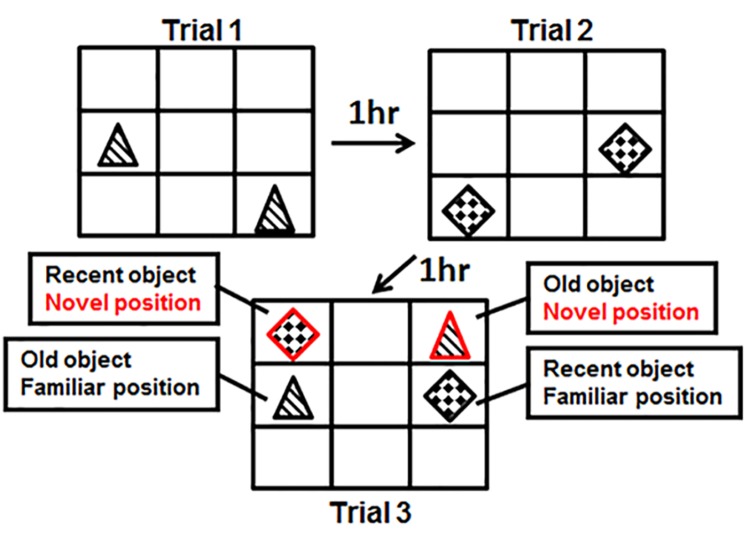
Depiction of the three test trials used in the stOR task. Animals were placed in the chamber with two objects (trial 1). After a 1-hour delay, animals were returned to the chamber with two new objects; after another 1-hour delay animals were returned to the chamber containing the four previously encountered objects. One object from trial 1 and one object from trial 2 were placed in new positions while the other objects remained in their original positions.

Rats were handled and dosed as previously described. Two cohorts of animals were used for each experiment (saline vs. CIE and adolescent vs. adult), resulting in an n = 12/treatment group. Following the 24-day washout period, animals (PND-70 and PND-30) were habituated to the testing apparatus (33cm L x 38cm W x 68cm H), devoid of objects, once a day for 5-min/session for 3 days. Animals were then habituated to the testing apparatus containing two pseudo-randomly selected objects once a day for 5 min per session/day for the following 2 days (these objects were not used in the following test trials). All objects were made of metal, glass, plastic or ceramic, and measured 11-17cm H x 7-8cm W. Multiple object placement combinations were used and all objects and locations were counterbalanced across treatment groups (see Fig 1 for an example of object placement across trials). Balancing of objects based on basal preferences and the effect of object location was minimized within the pilot studies. Time spent with an object regardless of position in the stOR pilot study was: 7.467–9.667 (seconds) ± 1.665–2.707 (SEM) within Trials 1 and 2. Testing box location within the room, handling of animals, noise attenuation, and lighting were all optimized in the same earlier pilot studies using male Sprague Dawley rats. White noise and ambient lighting was maintained (7–10 lux) throughout habituation and testing trials.

#### stOR testing

Trial 1—Animals were placed in the apparatus with two identical objects for 5 minutes and then immediately removed and placed in the home cage. All objects to which the animals were exposed on Trial 1 were subsequently referred to as ‘old objects’. Trial 2—After a 60 min delay, the animals were placed back in the apparatus with two novel objects in two new locations (subsequently referred to as ‘recent objects’) for 5 min and then returned to the home cage. Trial 3—After another 60 min delay the animals were placed back in the apparatus with the two ‘old objects’ and the two ‘recent objects’, one old object and one recent object remained in the same locations in which they had been previously placed (‘familiar position’) while the other objects were moved to new locations (‘novel position’). The animals were allowed to explore the arena for 5 min before being removed and placed back in their home cages. The object manipulations are depicted in Fig 1. Object placement and order was counterbalanced across cage mates and treatment groups. Use of the 60-minute delay was based on a study by Clark and colleagues [30]. Using various delays (10 seconds to 24 hours) and various hippocampal lesion paradigms they showed that the 60-minute delay was the most sensitive delay to uncover hippocampal deficits in the object recognition task.

All behavior was recorded using digital video software and subsequently analyzed by trained observers (AnyMaze, Stoelting, Chicago, IL, USA). Two independent raters reviewed and scored each video. The two individuals (H.S. and K.M.) were blind to the animals’ dose condition. Both raters were previously trained to confirm scoring accuracy by the study author (M-L.R.). Inter-rater agreement was assessed and scores were averaged across the two raters. Object exploration/inspection time was operationally defined as the time during which the animal was directing its nose and/or moving their vibrissae towards the object at a distance ≤1cm and/or touching the object with its nose or vibrissae. Running around the object, attempting to sit or climb on the object was not recorded as object exploration. Any animal that failed to interact with the objects during Test 1 or 2 was removed from the analysis.

Inspection behavior was expressed as position preference or a recency preference. Position preference was defined as the difference between the inspection time for objects in the novel position and the inspection time for objects in the familiar position, divided by the total inspection time (T_novel_−T_familiar_)/(T_total_)_._ Similarly, the recency preference was defined as the difference between the inspection time for recent objects and the inspection time for older objects, divided by the total inspection time (T_recent_−T_old_)/(T_total_). Total inspection time during the test phase was defined as the sum of time inspecting all objects in any position (T_total_). Using this value in the denominator serves to account for individual differences in general exploratory behavior.

### ChAT Immunohistochemistry

Twenty-five days after AIE (n = 6) or vehicle (n = 6) exposure, a separate group of animals was anesthetized with isoflurane and transcardially perfused with 0.1 M phosphate buffer saline (PBS; pH 7.4; 300 ml at 25 ml/min) followed by perfusion with 300 ml 4% paraformaldehyde (PFA; freshly prepared) in 0.1 M PB. The brains were post-fixed for 24 hours in 4.0% PFA at 4°C, followed by transfer to sucrose solution until they were sectioned. 40μm thick sections were cut coronally on a vibratome and stored in cryoprotectant (30% glycol/30% ethylene glycol in PBS) at -20°C. Free-floating sections (every 12th section containing the region of interest) were washed in 0.1 M PBS, incubated in 0.3% H2O2, and blocked with normal goat serum (MP Biomedicals, Solon, OH, USA). The sections were incubated in goat polyclonal anti-ChAT (1:800, AB144P; Millipore, Temecula, CA, USA) for 24 hours at 4°C. The sections were then washed with PBS, incubated with biotinylated secondary anti-goat antibody (1:200; Vector Laboratories, Burlingame, CA, USA) for one hour, and incubated in avidin-biotin complex solution (Vector ABC Kit; Vector Laboratories) for one hour. The chromagen, nickel-enhanced diaminobenzidine (DAB, Sigma-Aldrich, St. Louis MO, USA), was used to visualize immunoreactivity. Negative control for non-specific binding of the secondary antibody was conducted on separate sections employing the aforementioned procedures with the exception that the primary antibody was omitted.

The number of ChAT positive neurons was quantified by image analysis software as previously described [19]. Briefly, Bioquant Nova Advanced Image Analysis (R&M Biometric, Nashville, TN, USA) was used for image capture and analysis. Images were captured by using an Olympus BX50 Microscope and Sony DXC-390 video camera linked to a computer. For ChAT+IR (immunoreactivity), the ChAT positive neurons were counted within the region of interest and expressed as cells per square millimeter. Ch1 and Ch2 are contained in the medial septal nucleus (MS) and the nucleus of vertical limb of the diagonal band (VDB) respectively. Ch3 is mostly in the lateral portion of the horizontal limb nucleus of the diagonal band, and Ch4 is the nucleus basalis, and also parts of the diagonal band nuclei. For Ch1 and Ch2 sectors, coronal sections were from bregma 0.7 to 0.2 mm; for Ch3 and Ch4, from 0.48 to -0.30 mm. Both sides in every section were used, at least four to five sections for each brain, and the average value were used.

### Statistical Analysis

The stOR data was analyzed using hierarchical linear regression. This approach was chosen because it allows for making inferences about pre-treatment effects (saline vs. AIE) while controlling for object inspection time during the initial training trials. That is, regression allows us to determine the strength of the association between group membership and either recency or location preference. For example, a significant positive relationship would indicate that membership in the “AIE” class is associated with greater preference for the novel object (in the location preference) or the more recent object (in the recency preference). A significant negative relationship would indicate that membership in the “AIE” class is associated with greater preference for the familiar object (in the location preference) or the older object (in the recency preference). Importantly, this regression procedure allows us to determine these associations after controlling for individual differences in object inspection time during the initial learning trials by entering those data in the first step of the regression equation. Separate regressions were run for each dependent measure (object recency preference and object location preference). Trial 1 and Trial 2 inspection times were entered in the first step of the regression to control for initial object exposure. Group (experiment 1: saline v. AIE; experiment 2: adolescent v. adult) was entered on the second step. Model, R^2^ change (R^2^Δ), and beta coefficient statistics were calculated. Where the regression analyses were significant, we calculated and presented the predicted preference indices (i.e., the preference after correcting for individual differences in initial object inspection) as well as the observed preference indices. Independent Student’s *t*-tests were used to evaluate group differences on the predicted and observed preference indices. One-sample *t*-tests were used to determine if the group mean preference was significantly different from zero. Where the preference index is not different from zero, there is no demonstrable preference. Independent Student’s *t*-tests were used to assess differences in ChAT+IR in tissue from AIE- vs. vehicle-exposed animals. All analyses were conducted using SPSS (v.22; Chicago, IL, USA). Statistical significance was assessed using an alpha level of 0.05. All data are presented in figures as the mean +/- S.E.M.

## Results

### Selective Memory Deficits

#### stOR—temporal component

**Fig 2:**
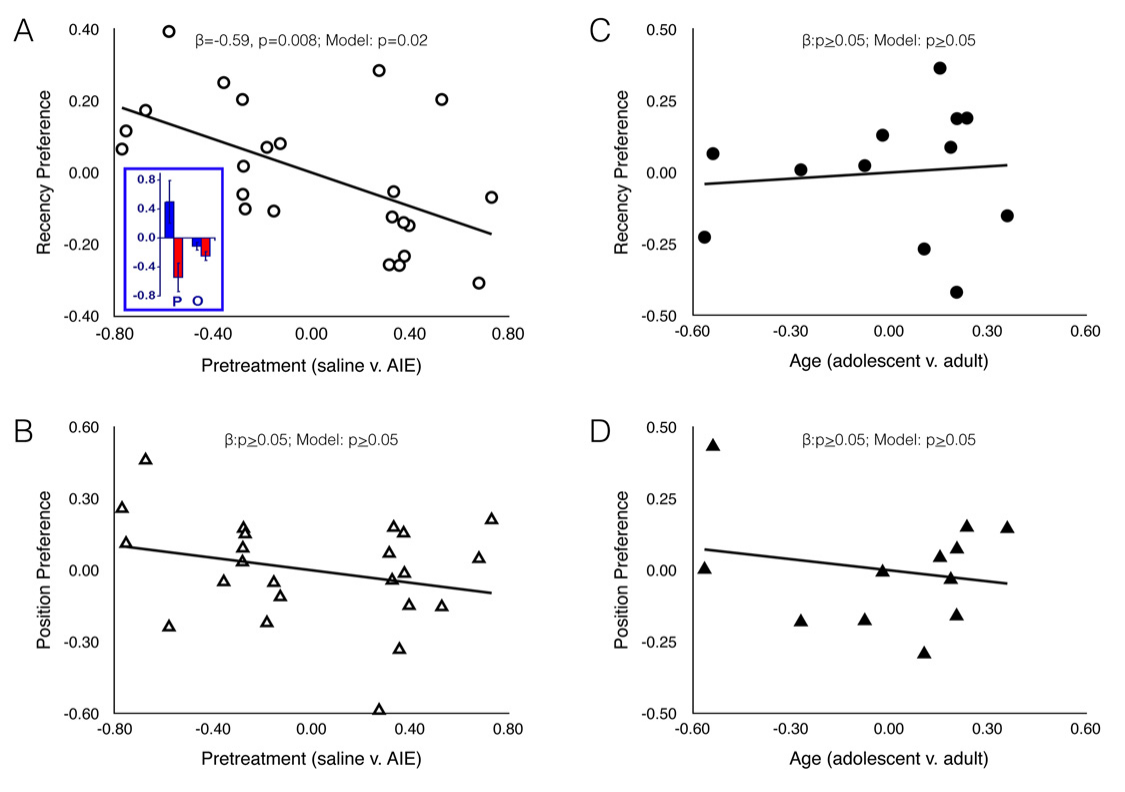
AIE produces deficits in object recognition memory in adulthood. Partial regression plots demonstrating the relationship between pre-treatment condition (saline v. AIE) and recency preference (panel A) and position preference (panel B); and between age (adolescent v. adult) and recency preference (panel C) and position preference (panel D). These plots represent the relationship between the independent variable (pre-treatment or age) and the dependent variable (recency preference or position preference) after controlling for time spent inspecting objects in Trials 1 and 2. Pretreatment was significantly predictive of the recency preference (panel A), but was not significantly predictive of position preference (panel B). Age was not predictive of recency or position preference (panels C and D). Panel A inset depicts the group mean (+/- SEM) predicted preference indices (derived from the regression model, denoted by “P”) and the observed preference indices based on uncorrected data (denoted by “O”).

Animals encountered two pairs of identical objects, having been exposed to one of those object pairs 1hr before (recent objects) and the other pair 2hrs before. In this paradigm, we expected AIE exposed animals to spend more time exploring the older objects versus the more recent objects, because the memory for the older objects had diminished relative to the memory for the more recently encountered objects. Results reveal that the complete model (Trial 1, Trial 2, and pretreatment group) accounts for a significant proportion of variance in recency preference (Adjusted R^2^ = 0.30, F(3,19) = 4.17, p = 0.02). Moreover, the addition of Group (AIE v. saline) in the second step significantly improves the predictive value of the model (R^2^Δ = 0.28, F(1,19) = 8.85, p = 0.008). That is, the AIE group is associated with a preference for the older objects after controlling for time spent inspecting those objects in Trials 1 and 2 (β = -0.59, t = -2.97, p = 0.008). This is illustrated in the partial regression plot (Fig 2A). The color inset in Fig 2 demonstrates the relative preference (+/- SEM) for the older object in the AIE group (red bars) and the control group (blue bars). The left side of that inset shows the mean preference based on predicted scores (P; after correcting for individual differences in object inspection during the learning trials). The right side of that inset shows the mean preference based on observed scores (O), which were not corrected for individual differences in object inspection during the learning trials.

Analysis of predicted preference scores (i.e., after correcting for individual differences in object inspection during the learning trials) from the inset reveals that AIE pretreated animals (red bar denoted “P”) spend significantly more time inspecting the old objects than do control pretreated animals (blue bar denoted “P”; t(21) = 2.88, p≤0.005). Indeed, the preference for old objects that the model predicted for AIE pretreated animals was significantly different from zero (t(10) = -2.74, p = 0.01) while no preference was observed among the control animals, i.e., their preference scores were not significantly different from zero (t(11) = 1.69, p = 0.06). Analysis of observed (i.e. uncorrected) preference scores revealed no significant difference between AIE (red bar denoted “O”) and control (blue bar denoted “O”; t(21) = 1.63, p = 0.06). Interestingly, when no correction is made for individual differences in initial object inspection during the learning trials, there is a significant preference for older objects (relative to zero) in both AIE (t(10) = -4.07, p = 0.001) and control (t(11) = -2.02, p = 0.04) animals.

#### stOR- spatial component

Using this task, our expectation was that control animals would show a greater preference for the object in the novel position than would AIE pre-treated animals. This was not the case. The complete model (Trial 1, Trial 2, and pretreatment group) did not account for a significant proportion of variance in object location preference (Adjusted R^2^ = 0.096, F(3,19) = 1.78, p = 0.19); and the addition of Group (AIE vs. saline) in the second step did not significantly improve the predictive value of the model (R^2^Δ = 0.06, F(1,19) = 1.51, p = 0.24; Fig 1B). This indicates that memory for the location of the objects (novel versus familiar) was not affected by the pre-treatment condition (ethanol vs. control).

Because some studies have suggested that AIE promotes the perpetuation of adolescent-like characteristics into adulthood [22], [31], we conducted a second experiment with a separate cohort of animals to determine if the deficits observed after AIE were reminiscent of immaturity we compared treatment-naïve adult and adolescent animals in the same stOR task. Analyses were conducted in the same fashion as describe above (see Fig 1C and 1D). The complete model (Trial 1, Trial 2, and age group) did not account for a significant proportion of variance in recency preference (Adjusted R^2^ = -0.20, F(3,8) = 0.38, p = 0.77); and the addition of Group (adolescent v. adult) in the second step did not significantly improve the predictive value of the model (R^2^Δ = 0.04, F(1,8) = 0.34, p = 0.57). Similar results were obtained for object location preference: the complete model (Trial 1, Trial 2, and age group) did not account for a significant proportion of variance in object location preference (Adjusted R^2^ = -0.34, F(3,8) = 0.07, p = 0.98); and the addition of Group (adolescent v. adult) in the second step did not significantly improve the predictive value of the model (R^2^Δ = 0.009, F(1,8) = 0.08, p = 0.79).

### ChAT Staining in Areas Ch1-4

**Fig 3:**
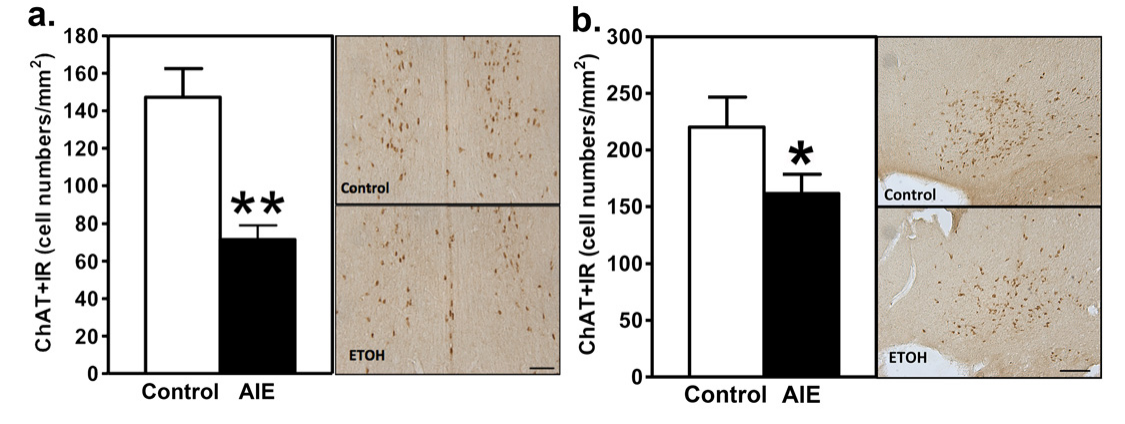
AIE decreases ChAT+IR in the basal forebrain in adulthood. AIE decreases ChAT+IR in the Ch1 and Ch2 nuclei of the basal forebrain of adult rats (a). Left Panel—the cell density of ChAT+IR is significantly decreased in the Ch1 and Ch2 nuclei at 0.70 ~ 0.20 mm from bregma 25 days after AIE, **p<0.01. Right Panel—representative photomicrography ChAT+IR neurons in the Ch1 and Ch2 nuclei from a control animal (Control), and an AIE-exposed animal (ETOH). AIE decreases ChAT+IR in the Ch3 and Ch4 nuclei of the basal forebrain of adult rats (b). Left Panel—the cell density of ChAT+IR is significantly decreased in the Ch3 and Ch4 nuclei at 0.48 ~ 0.40 mm from bregma 25 days after AIE, *p = 0.046. Right Panel—representative photomicrography ChAT+IR neurons in the Ch3 and Ch4 nuclei from a control animal (Control), and an ethanol-exposed animal (ETOH). Scale bar = 50 μm.

As Fig 2 illustrates, AIE decreased ChAT+IR in the medial septum and vertical limb of the diagonal band of Broca (Ch1 and Ch2) nuclei of the basal forebrain of adult rats 25 days after the termination of AIE. Specifically, ChAT+IR cell density was decreased by approximately 50% (p<0.01) in cholinergic Ch1 and Ch2 nuclei. This is illustrated quantitatively in the left panel of Fig 2, with visualization of ChAT+IR neurons in the right panel. We also assessed diagonal band/nucleus basalis (Ch3-4) ChAT+IR neurons and found 220±26 (SEM) and 161±17 (SEM) ChAT+IR neurons/mm^2^ control and AIE animals, respectively (p = 0.046; Fig 3). These findings indicate AIE leads to a persistent loss of adult ChAT+ neurons, consistent with previous studies [19,20].

## Discussion

The long-term impact of repeated ethanol exposure during adolescence on neurobehavioral function in adulthood has become a topic of intensive interest and recent investigation. Because acute ethanol is well known to affect memory and hippocampal function, and to do so more potently during adolescence than adulthood [31,32], the enduring effects of AIE on memory is of particular interest. In this study we found that AIE impaired object recognition memory in the stOR task. Specifically, we found that adult animals that had been exposed to AIE during adolescence manifested deficits in recalling information over time, independent of their spatial location. In addition, naïve adolescent and adult animals performed similarly on the task, suggesting that the AIE-induced memory deficits were not related to behavioral immaturity on the part of AIE exposed adult animals. Finally, consistent with previous studies [19,20], AIE significantly reduced the density of ChAT-positive neurons on forebrain areas Ch1-2, which project widely to the hippocampal formation and influence memory-related hippocampal function, as well as in areas Ch3-4.

Although the persistence of adolescent-typical characteristics in adulthood has been observed in electrophysiological studies and behavioral studies across multiple laboratories and multiple strains using varying adolescent exposure regimens (see [31] for review), that mechanism does not appear to have driven the behavioral effects of AIE in this study. Thus there is selectivity in the persistence of certain immature characteristics in adulthood after AIE, and possibly a propensity for hippocampal vulnerability to such changes. For example, the reduction in tonic inhibition in the dentate gyrus after AIE is reminiscent of adolescence [33]. While greater behavioral disinhibition in the open field conflict task [34] and adolescent-typical behavioral responses in the context-dependent (and hippocampal-dependent) fear conditioning task [13,35] are also observed after AIE, they are not present in other aspects of those tasks or others. Additional studies are needed in order to more fully explore the neural and behavioral domains in which AIE induces the persistence of adolescent-like characteristics into adulthood.

The functional relevance of the effects of AIE on adult hippocampal physiology, morphology, and synaptic organization that we have reported recently [22] is consistent with the present findings that AIE disrupted memory in the stOR task. Although we observed AIE-induced memory impairment for the temporal, but not the spatial component of the stOR task, the fact that this effect was observed on a task that included both spatial and temporal components is consistent with previous studies in which AIE had little effect on simple spatial learning tasks [9,11], but markedly impaired performance under challenged or challenging conditions [9,11,17]. Clarke and colleagues [36] have shown that, in the novel object recognition task, memory consolidation is marked by transient hippocampal potentiation and a short de-potentiation phase necessary for reconsolidation. This vulnerable reconsolidation phase is an important aspect of the stOR task used in the present study since it is required every time new objects or new object locations are introduced, thus allowing this novel information to be added to the previously stored memory. This continuing need to reconsolidate memory about ‘what’, ‘where’, and ‘when’ makes this task more cognitively demanding than the traditional novel object recognition task. The cognitive challenge of this ‘layering of information’ in the stOR task may be what distinguished it from other less challenging tasks on which AIE animals perform as well as controls, such as the version of the radial arm maze task that we have used previously [11].

The fact that hippocampal networks must potentiate and de-potentiate relatively rapidly for novel object recognition learning to occur [36], suggests that the effects of AIE on LTP induction that we have observed previously [22] could underlie the parallel effects of AIE on memory in the stOR task. If, as we have suggested previously [22], AIE induces a state of hippocampal hyperplasticity that may transiently occlude subsequent manifestations of plasticity, then it is possible that the affected hippocampal circuits would not be capable of the rapid shifts between potentiation and de-potentiation required for the learning to occur. Put another way, the facilitated induction of LTP in the hippocampus after AIE [22] could render the circuits less “nimble” in their capacity to engage in rapid sequences of potentiation and de-potentiation, thus compromising the memory consolidation and reconsolidation needed for stOR learning to occur optimally. Alternatively, since reconsolidation is necessary for complete information retrieval in Trial 3, the information presented in Trial 2 could interfere with the retention and/or retrieval of information from Trial 1 in animals with hyperplastic hippocampal circuits.

Previous studies have shown reductions of the density of ChAT+ neurons in the Ch1-4 regions of the forebrain [19,20,37], thus indicating that AIE affects cholinergic neurons throughout the extent of the Ch1 through Ch4 regions. We observed similar effects in the present study, suggesting persistently diminished hippocampal cholinergic signaling after AIE. This finding provides an important replication of the previous studies, using our specific AIE parameters. Although this finding does not establish a causal relationship between AIE-induced decreases in forebrain cholinergic neurons and the memory deficits we observed, their co-occurrence is informative because cholinergic neurons in Ch1-2 project diffusely into the hippocampal formation, drive the activity of both primary and inhibitory neurons throughout the structure, and modulate circuit function [38]. In addition, a decrease in the cholinergic projections from Ch-3 to the entorhinal cortex would also be expected to compromise hippocampal function. However, it should be noted that Ch-3 also projects to the pyriform cortex and olfactory bulbs and Ch-4 projects widely to the neocortex. Thus the effect of AIE on cholinergic marker in those regions would be expected to influence cholinergic function beyond the hippocampal formation.

Although it appears that AIE compromises cholinergic projections rather broadly, it is important to note that cholinergic projections from the forebrain are known to regulate hippocampal neurogenesis [39–41]. Forebrain cholinergic neurons have monosynaptic connections with dentate gyrus neuroprogenitors [41], and AIE, but not adult CIE, causes a persistent loss of hippocampal neurogenesis [42]. Although the role of neurogenesis in hippocampal function is poorly understood it is implicated in complex components of learning as well as negative affect. In contrast to non-specific lesions of the Ch1-2 region, selective lesions of cholinergic neurons in that region, as found after AIE, often do not result in robust learning and memory deficits [43], but have been shown to induce learning deficits that are evident as task complexity is increased [44,45], consistent with the present finding of deficits in stOR memory. More work will be required to determine whether AIE-induced depletion of Ch1-2 cholinergic neurons bears a causal relationship to the stOR deficits we have observed or the deficits observed previously on long-delay temporal object recognition [17]. However, the present results do suggest that AIE-induced deficits in hippocampally mediated behavioral deficits might be ameliorated by acute treatment with agents that increase cholinergic function. In addition, since the AIE-induced reduction of Ch1-2 cholinergic neuron density is likely to be initiated during the AIE exposure period, it is possible that cholinergic replacement during that time would mitigate the effects of the chronic deprivation of hippocampal circuits from their normal cholinergic inputs, thereby mitigating the long-term effects of AIE on hippocampal function.

In conclusion, the present findings identify a specific deficit in object recognition memory after AIE. This deficit was accompanied by a decrease in the density of cholinergic neurons that are known to project to the hippocampal formation and modulate learning-related circuit function. These findings add to a growing literature indicating that intermittent ethanol exposure during adolescence results in neurobehavioral deficits in the domain of memory and memory-related function that persist into adulthood.

## References

[1] Walker DW, Hunter BE (1978) Short-term memory impairment following chronic alcohol consumption in rats. Neuropsychologia 16: 545–553. 569777 10.1016/0028-3932(78)90082-9

[2] Riley JN, Walker DW (1978) Morphological alterations in hippocampus after long-term alcohol consumption in mice. Science 201: 646–648. 566953 10.1126/science.566953

[3] Walker DW, Barnes DE, Zornetzer SF, Hunter BE, Kubanis P (1980) Neuronal loss in hippocampus induced by prolonged ethanol consumption in rats. Science 209: 711–713. 7394532 10.1126/science.7394532

[4] Swartzwelder HS, Wilson WA, Tayyeb MI (1995) Age-dependent inhibition of long-term potentiation by ethanol in immature versus mature hippocampus. Alcohol Clin Exp Res 19: 1480–1485. 8749814 10.1111/j.1530-0277.1995.tb01011.x

[5] Little PJ, Kuhn CM, Wilson WA, Swartzwelder HS (1996) Differential effects of ethanol in adolescent and adult rats. Alcohol Clin Exp Res 20: 1346–1351. 8947309 10.1111/j.1530-0277.1996.tb01133.x

[6] Markwiese BJ, Acheson SK, Levin ED, Wilson WA, Swartzwelder HS (1998) Differential effects of ethanol on memory in adolescent and adult rats. Alcohol Clin Exp Res 22: 416–421. 9581648

[7] Brown SA, McGue M, Maggs J, Schulenberg J, Hingson R, et al (2008) A developmental perspective on alcohol and youths 16 to 20 years of age. Pediatrics 121 Suppl 4: S290–310. 10.1542/peds.2007-2243D 18381495 PMC2765460

[8] Matthews DB (2010) Adolescence and alcohol: recent advances in understanding the impact of alcohol use during a critical developmental window. Alcohol 44: 1–2. 10.1016/j.alcohol.2009.10.018 20113869

[9] White AM, Ghia AJ, Levin ED, Swartzwelder HS (2000) Binge pattern ethanol exposure in adolescent and adult rats: differential impact on subsequent responsiveness to ethanol. Alcohol Clin Exp Res 24: 1251–1256. 10968665

[10] Vetreno RP, Crews FT (2012) Adolescent binge drinking increases expression of the danger signal receptor agonist HMGB1 and Toll-like receptors in the adult prefrontal cortex. Neuroscience 226: 475–488. 10.1016/j.neuroscience.2012.08.046 22986167 PMC3740555

[11] Risher ML, Fleming RL, Boutros N, Semenova S, Wilson WA, et al (2013) Long-term effects of chronic intermittent ethanol exposure in adolescent and adult rats: radial-arm maze performance and operant food reinforced responding. PLoS One 8: e62940 10.1371/journal.pone.0062940 23675442 PMC3652810

[12] Sircar R, Sircar D (2005) Adolescent rats exposed to repeated ethanol treatment show lingering behavioral impairments. Alcohol Clin Exp Res 29: 1402–1410. 16131847 10.1097/01.alc.0000175012.77756.d9

[13] Broadwater M, Spear LP (2013) Consequences of ethanol exposure on cued and contextual fear conditioning and extinction differ depending on timing of exposure during adolescence or adulthood. Behav Brain Res 256: 10–19. 10.1016/j.bbr.2013.08.013 23938333 PMC3816365

[14] Pascual M, Blanco AM, Cauli O, Minarro J, Guerri C (2007) Intermittent ethanol exposure induces inflammatory brain damage and causes long-term behavioural alterations in adolescent rats. European Journal of Neuroscience 25: 541–550. 17284196 10.1111/j.1460-9568.2006.05298.x

[15] Silvers J, Tokunaga S, Mittleman G, Matthews D (2003) Chronic intermittent injections of high-dose ethanol during adolescence produce metabolic, hypnotic, and cognitive tolerance in rats. Alcoholism, Clinical and Experimental Research 27: 1606–1612. 14574231 10.1097/01.ALC.0000090141.66526.22

[16] Silvers JM, Tokunaga S, Mittleman G, O'Buckley T, Morrow AL, et al (2006) Chronic intermittent ethanol exposure during adolescence reduces the effect of ethanol challenge on hippocampal allopregnanolone levels and Morris water maze task performance. Alcohol 39: 151–158. 17127134 10.1016/j.alcohol.2006.09.001

[17] Vetreno RP, Yaxley R, Paniagua B, Crews FT (2015) Diffusion tensor imaging reveals adolescent binge ethanol-induced brain structural integrity alterations in adult rats that correlate with behavioral dysfunction. Addict Biol. 12232. 10.1111/adb.12232 PMC4532660 25678360

[18] Vetreno RP, Crews FT (2015) Binge ethanol exposure during adolescence leads to a persistent loss of neurogenesis in the dorsal and ventral hippocampus that is associated with impaired adult cognitive functioning. Front Neurosci 9: 35 10.3389/fnins.2015.00035 25729346 PMC4325907

[19] Vetreno RP, Broadwater M, Liu W, Spear LP, Crews FT (2014) Adolescent, but not adult, binge ethanol exposure leads to persistent global reductions of choline acetyltransferase expressing neurons in brain. PLoS One 9: e113421 10.1371/journal.pone.0113421 25405505 PMC4236188

[20] Boutros N, Semenova S, Liu W, Crews FT, Markou A (2014) Adolescent intermittent ethanol exposure is associated with increased risky choice and decreased dopaminergic and cholinergic neuron markers in adult rats. Int J Neuropsychopharmacol 18: 2. 10.1093/ijnp/pyu003 PMC4368879 25612895

[21] Kanju PM, Parameshwaran K, Sims-Robinson C, Uthayathas S, Josephson EM, et al (2012) Selective cholinergic depletion in medial septum leads to impaired long term potentiation and glutamatergic synaptic currents in the hippocampus. PLoS One 7: e31073 10.1371/journal.pone.0031073 22355337 PMC3280283

[22] Risher ML, Fleming RL, Risher WC, Miller KM, Klein RC, et al (2015) Adolescent intermittent alcohol exposure: persistence of structural and functional hippocampal abnormalities into adulthood. Alcohol Clin Exp Res 39: 989–997. 10.1111/acer.12725 25916839 PMC4452443

[23] Acheson SK, Bearison C, Risher ML, Abdelwahab SH, Wilson WA, et al (2012) Effects of acute or chronic ethanol exposure during adolescence on behavioral inhibition and efficiency in a modified water maze task. PloS one 8: 1–15. 10.1371/journal.pone.0077768 PMC3798405 24147077

[24] Squeglia LM, Schweinsburg AD, Pulido C, Tapert SF (2011) Adolescent binge drinking linked to abnormal spatial working memory brain activation: differential gender effects. Alcohol Clin Exp Res 35: 1831–1841. 10.1111/j.1530-0277.2011.01527.x 21762178 PMC3183294

[25] Spear LP, Swartzwelder HS (2014) Adolescent alcohol exposure and persistence of adolescent-typical phenotypes into adulthood: A mini-review. Neurosci Biobehav Rev 45: 1–8. 10.1016/j.neubiorev.2014.04.012 24813805 PMC4134704

[26] Kart-Teke E, De Souza Silva MA, Huston JP, Dere E (2006) Wistar rats show episodic-like memory for unique experiences. Neurobiol Learn Mem 85: 173–182. 16290193 10.1016/j.nlm.2005.10.002

[27] Ennaceur A, Neave N, Aggleton JP (1997) Spontaneous object recognition and object location memory in rats: the effects of lesions in the cingulate cortices, the medial prefrontal cortex, the cingulum bundle and the fornix. Exp Brain Res 113: 509–519. 9108217 10.1007/pl00005603

[28] Mitchell JB, Laiacona J (1998) The medial frontal cortex and temporal memory: tests using spontaneous exploratory behaviour in the rat. Behav Brain Res 97: 107–113. 9867236 10.1016/s0166-4328(98)00032-1

[29] Ennaceur A, Delacour J (1988) A new one-trial test for neurobiological studies of memory in rats. 1: Behavioral data. Behav Brain Res 31: 47–59. 3228475 10.1016/0166-4328(88)90157-x

[30] Clark RE, Zola SM, Squire LR (2000) Impaired recognition memory in rats after damage to the hippocampus. J Neurosci 20: 8853–8860. 11102494 10.1523/JNEUROSCI.20-23-08853.2000 PMC6773055

[31] Spear LP, Swartzwelder HS (2014) Adolescent alcohol exposure and persistence of adolescent-typical phenotypes into adulthood: a mini-review. Neurosci Biobehav Rev 45: 1–8. 10.1016/j.neubiorev.2014.04.012 24813805 PMC4134704

[32] White AM, Swartzwelder HS (2004) Hippocampal function during adolescence: a unique target of ethanol effects. Ann N Y Acad Sci 1021: 206–220. 15251891 10.1196/annals.1308.026

[33] Fleming RL, Li Q, Risher ML, Sexton HG, Moore SD, et al (2013) Binge-Pattern Ethanol Exposure During Adolescence, but Not Adulthood, Causes Persistent Changes in GABA(A) Receptor-Mediated Tonic Inhibition in Dentate Granule Cells. Alcohol Clin Exp Res 36: 279–285. 10.1111/acer.12087 PMC3754782 23413887

[34] Desikan A, Wills DN, Ehlers CL (2014) Ontogeny and adolescent alcohol exposure in Wistar rats: open field conflict, light/dark box and forced swim test. Pharmacol Biochem Behav 122: 279–285. 10.1016/j.pbb.2014.04.011 24785000 PMC4105136

[35] Broadwater MA, Spear LP (2014) Tone conditioning potentiates rather than overshadows context fear in adult animals following adolescent ethanol exposure. Dev Psychobiol 56: 1150–1155. 10.1002/dev.21186 24339140

[36] Clarke JR, Cammarota M, Gruart A, Izquierdo I, Delgado-Garcia JM (2010) Plastic modifications induced by object recognition memory processing. Proceedings of the National Academy of Sciences of the United States of America 107: 2652–2657. 10.1073/pnas.0915059107 20133798 PMC2823877

[37] Ehlers CL, Criado JR, Wills DN, Liu W, Crews FT (2011) Periadolescent ethanol exposure reduces adult forebrain ChAT+IR neurons: correlation with behavioral pathology. Neuroscience 199: 333–345. 10.1016/j.neuroscience.2011.10.011 22033458 PMC3264427

[38] Teles-Grilo Ruivo LM, Mellor JR (2013) Cholinergic modulation of hippocampal network function. Front Synaptic Neurosci 5: 2 10.3389/fnsyn.2013.00002 23908628 PMC3726829

[39] Mohapel P, Leanza G, Kokaia M, Lindvall O (2005) Forebrain acetylcholine regulates adult hippocampal neurogenesis and learning. Neurobiol Aging 26: 939–946. 15718053 10.1016/j.neurobiolaging.2004.07.015

[40] Kaneko N, Okano H, Sawamoto K (2006) Role of the cholinergic system in regulating survival of newborn neurons in the adult mouse dentate gyrus and olfactory bulb. Genes Cells 11: 1145–1159. 16999735 10.1111/j.1365-2443.2006.01010.x

[41] Vivar C, Potter MC, Choi J, Lee JY, Stringer TP, et al (2012) Monosynaptic inputs to new neurons in the dentate gyrus. Nat Commun 3: 1107 10.1038/ncomms2101 23033083 PMC4603575

[42] Broadwater MA, Liu W, Crews FT, Spear LP (2014) Persistent loss of hippocampal neurogenesis and increased cell death following adolescent, but not adult, chronic ethanol exposure. Dev Neurosci 36: 297–305. 10.1159/000362874 24993092 PMC4125431

[43] Fletcher BR, Baxter MG, Guzowski JF, Shapiro ML, Rapp PR (2007) Selective cholinergic depletion of the hippocampus spares both behaviorally induced Arc transcription and spatial learning and memory. Hippocampus 17: 227–234. 17286278 10.1002/hipo.20261

[44] Lehmann O, Grottick AJ, Cassel JC, Higgins GA (2003) A double dissociation between serial reaction time and radial maze performance in rats subjected to 192 IgG-saporin lesions of the nucleus basalis and/or the septal region. Eur J Neurosci 18: 651–666. 12911761 10.1046/j.1460-9568.2003.02745.x

[45] Cabrera SM, Chavez CM, Corley SR, Kitto MR, Butt AE (2006) Selective lesions of the nucleus basalis magnocellularis impair cognitive flexibility. Behav Neurosci 120: 298–306. 16719694 10.1037/0735-7044.120.2.298

